# Angiopoietin-2 binds to FGFR2, inhibits FGF-FGFR2 signaling, and delays cutaneous wound healing by inhibiting wound angiogenesis

**DOI:** 10.1007/s10456-025-09988-2

**Published:** 2025-08-05

**Authors:** Minji Sim, Hidetaka Ohnuki, Stewart Durell, Haydar Bulut, Yuyi Wang, Marzena Dyba, Sergey G. Tarasov, Lisa M. Jenkins, Giovanna Tosato

**Affiliations:** 1 Laboratory of Cellular Oncology, Center for Cancer Research, National Cancer Institute, National Institutes of Health, Bethesda, MD 20892 USA; 2 Laboratory of Cell Biology, Center for Cancer Research, National Cancer Institute, National Institutes of Health, Bethesda, MD 20892 USA; 3 Experimental Retrovirology Section, HIV & AIDS Malignancy Branch, Center for Cancer Research, National Cancer Institute, National Institutes of Health, Bethesda, MD 20892 USA; 4 Biophysics Resource, Center for Structural Biology, National Cancer Institute, Frederick, MD 21702 USA

**Keywords:** Angiogenesis, Wound healing, Angiopoietin2, FGF receptors

## Abstract

Wound healing is an essential repair process, and impaired wound healing is a common and sometimes debilitating medical problem. Despite advances in wound healing approaches, optimal management strategies are lacking, partly due to an incomplete understanding of the complex pathophysiology of this process. Here we show that Ang2, a previously known ligand for the Tie2 receptor, also binds to fibroblast growth factor receptor 2 (FGFR2) independently of Tie2 and attenuates FGF/FGFR2 signaling in endothelial cells. Functionally, Ang2 inhibits endothelial cell migration induced by FGF. In mouse, topical Ang2 delays the healing of skin wounds, associated with reduced wound angiogenesis and recruitment of mesenchymal-type cells. Additionally, topical AMG386, a blocker of Ang1/Ang2 binding to Tie2 and systemic REGN910, a blocker of Ang2 binding to Tie2, accelerate wound repair, associated with increased wound angiogenesis and recruitment of inflammatory cells. These results identify the tyrosine kinase FGFR2 as a previously unrecognized Ang2 receptor, explaining some of the context-dependent functions of Ang2 in endothelial cells. Since Ang2 is induced in cutaneous wounds and endogenous FGF/FGFR2 is essential for wound repair, Ang2 blockade holds promise as a new evidence-based therapeutic option to promote wound repair.

**Supplementary Information:**

The online version contains supplementary material available at 10.1007/s10456-025-09988-2.

## Introduction

Tissue repair is one of the most complex biological processes that is activated after an injury [1, 2]. In mammals, the normal response to skin injury occurs in distinct yet overlapping stages that involve many cell types undergoing marked changes in gene expression, signaling and phenotype leading to cell migration, proliferation, and differentiation. If successful, these processes stop in a precise sequence as recovery progresses [3]. Normal repair of acute skin wounds starts with activation of the coagulation cascade and inflammatory pathways that limit losses of blood and body fluids, prevent infection, and remove dead tissue. The next stage is characterized by new tissue formation through blood capillary sprouting and neovascularization, migration of mesenchymal-type cells and other cell types, and production of extracellular matrix components to form granulation tissue [4, 5]. This represents a scaffold for cell migration, proliferation, and differentiation by keratinocytes and other cell types participating in the tissue repair process to restore the barrier function of the epithelium, followed by a progressive shutdown of the processes activated after injury [6].

Skin injuries are common, and comorbidities from the increasingly aging and obese population have led to a growing demand for wound closure products, with an estimated market size of 21.4 billion in 2022 (G.V. Research, “Wound care Market Size, Share & Trends Analysis Report by Product” (2023)). Advances have been made in the care of wounds and many new products are in the clinical pipeline [7]. However, to move beyond current non-specific wound-care products and accelerate development of evidence-based targeted products, an improved fundamental understanding of the molecular events that drive wound repair is needed.

Angiogenesis, the process of new vessel formation, is an essential component of wound repair, driven by VEGF, FGF, PDGF and other effectors [8]. Genetic, epigenetic, biochemical, and functional studies have profiled key aspects of angiogenesis. Much of this research has focused on vascular development, adult physiology, and angiogenesis in the context of cancer and cardiovascular disease [9–16]. However, key regulators of angiogenesis operate in a context-dependent manner such that lessons learned from one setting may not apply to other settings. Angiopoietin-2 (Ang2), a ligand of the endothelial Tie2 tyrosine kinase receptor, is a context-dependent agonist or antagonist of Tie2 [18–21]. In cancer, Ang2 promotes tumor angiogenesis, but in settings of inflammation Ang2 destabilizes the vasculature [21].

Ang2 is locally induced during skin wound healing [22, 23], but its functions in this context are not well understood. In this study, we have identified Ang2 as a previously unrecognized ligand for FGFR2 that inhibits FGF/FGFR2 signaling. In cutaneous wounds, Ang2 delays wound repair and reduces wound angiogenesis. Blocking Ang2 with topical AMG386 accelerates wound healing.

## Materials and methods

### Cells and cell culture

The human umbilical vein endothelial cells (HUVEC, ATCC, No. CRL-1730) were propagated as described [24]. HEK293T cells (ATCC, No. CRL-3216) were cultured in DMEM medium with 10% fetal bovine serum (Sigma, No. F2442) and penicillin/streptomycin. When indicated, HUVEC were incubated in starvation medium EBM-2 (Lonza, No. CC-3156) with Ascorbic acid (Lonza, No. CC-4116 A), Hydrocortisone (Lonza, No. CC-4112 A), and GA-1000 (Lonza, No. CC-4381). When indicated, HEK293T cells were incubated in starvation DMEM medium without FBS.

## Immunoblotting and Immunoprecipitation

HUVEC were incubated in starvation medium overnight unless otherwise specified. HEK293T cells were incubated in starvation medium overnight. Cells were then incubated with 100–1000 ng/ml recombinant Ang2 (expressed with a C-terminal His-tag in CHO cells; Biolegend, No. 753106) or/and 3 or 5 nM FGF1 (R&D Systems, No. 232-FA) for 5 min, and/or 314.8 nM (10 µg/ml) AMG386 (obtained under material CRADA from Amgen). Cells were washed with PBS containing 100 µM Na_3_VO_4_ and lysates were prepared with 1 x LDS sample buffer (ThermoFisher Scientific, No. NP0007) with 20 mM NaF and 10 mM pyrophosphate or RIPA buffer with 1 mM Na_3_VO_4_, phosphatase and protease inhibitor cocktail (ThermoFisher Scientific, No. 78442). Binding of Ang2 to FGFR2ß (IIIb)-Fc (R&D Systems 665-FR), to FGFR2α (IIIc)-Fc (R&D Systems, No. 712-FR) or hIgG-Fc (R&D Systems, No. 110-HG) was assessed using the previously described binding buffer [25] for 1 h. Immunoprecipitation was performed using proteinG-coated Dynabeads (ThermoFisher Scientific, No. 10004D) or with NTA-coated Dynabeads for His-Tag protein pulldown (ThermoFisher Scientific, No. 10103D). Following washes, complexes were eluted in 20 µl elution buffer (0.1 M Citrate pH 2–3 in distilled water). Precipitates and protein lysates were separated by SDS/PAGE using NuPage 4–12% Bis-Tris gels (ThermoFisher Scientific, No. NP0322) with MOPS running buffer. Proteins were transferred to nitrocellulose or PVDF membranes (ThermoFisher Scientific, No. 1B24002). Membranes were incubated overnight at 4 ºC with anti-pFGFR antibody (Cell Signaling Technology, No. 3471), anti-pEphrinB antibody (Cell Signaling Technology, No. 3481), anti-ephrinB2 antibody (Abcam, No. 131536), anti-pErk antibody (Cell Signaling Technology, No. 9101), anti-Erk antibody (Cell Signaling Technology, No. 4695), anti-pSTAT1 antibody (Cell Signaling Technology, No. 9167), anti-STAT1 antibody (Cell Signaling Technology, No. 9172), anti-pSTAT3 antibody (Cell signaling, No. 9145), anti-STAT3 antibody (Cell Signaling Technology, No. 9139), anti-pAKT antibody (Cell Signaling Technology, No. 4058), anti-AKT antibody (Cell Signaling Technology No. 9272), anti-pTie2 antibody (R&D Systems, No. AF2720), anti-Tie2 (R&D Systems, No. AF313), anti-Actin (Santa Cruz Biotechnology, No. sc-47778), anti-Ang2 (Santa Cruz Biotechnology, No. sc74403), anti-FGF1 antibody (Santa Cruz Biotechnology, No. sc55520), anti-FGFR2 (IIIß) antibody (R&D Systems, No. MAB665), anti-hIgG-Fcγ (Jackson ImmunoResearch, No. 109-005-008). After washing with TBST (K.D Medical Inc., RGE-3385), membranes were incubated with the appropriate HRP-linked secondary antibody, anti-rabbit IgG (Cell Signaling Technology, No. 7074), anti-mouse IgG (GE HealthCare, No. NA931V), anti-Goat IgG (R&D Systems, No. HAF109).

## LC-MS-MS

LC-MS/MS was performed essentially as described [26]. Briefly, dried peptides were solubilized in 2% acetonitrile, 0.5% acetic acid, 97.5% water for mass spectrometry analysis. Peptides were trapped on a trapping column and separated on a 75 μm x 15 cm, 2 μm Acclaim PepMap reverse phase column (Thermo Scientific, 16–494-6) using an UltiMate 3000 RSLCnano HPLC (Thermo Scientific). Peptides were separated at a flow rate of 300 nL/min followed by online analysis by tandem mass spectrometry using a Thermo Orbitrap Fusion mass spectrometer. Peptides were eluted into the mass spectrometer using a linear gradient from 96% mobile phase A (0.1% formic acid in water) to 55% mobile phase B (0.1% formic acid in acetonitrile). Proteome Discoverer 2.4 (Thermo) was used to search the data against human proteins from the UniProt database using SequestHT. Enriched proteins were identified by comparing the number of peptide spectral matches across samples. Pathway enrichment analysis (Ingenuity Pathway Analysis) was applied to the 368 Ang2-proximal proteins identified by LC-MC-MS.

## Predictive structure modeling

Models of ANG2/FGF1/FGFR2 complexes were predicted with AlphaFold 2.3.1 using the Multimer protocol [27] and (Evans R. et al., 2022, 10.1101/2021.10.04.463034). The image of the protein complex was prepared with Chimera 1.17.1 [28]. Models of ANG2/FGF1 or FGF2/FGFR2 complexes were predicted with AlphaFold3 [29]. Models of Ang2/FGF1/FGFR2 in complex with AMG386 were predicted using AlphaFold3 [29]. Sequences for Ang2, FGF1, FGFR2, and AMG386 were submitted to the server in multimer mode. Confidence scores were assessed using pLDDT values, with very high confidence defined as pLDDT > 90 (Suppl. Figure 1A-D). We further compared the predicted structures of FGFR2 (pink) and FGF1 (green to the known crystal structure of the FGFR2-FGF1 complex (PDB ID 1DJS, black). Additionally, the X-ray crystal structure of Ang2 (PDB ID 1Z3S) was superimposed onto the predicted Ang2 structure. The root-mean-square deviation (RMSD) values were 1.73 Å for 1DJS and 0.58 Å for 1Z3S, indicating a high degree of structural similarity between the predicted models and the experimental structures. To further validate the predicted interactions, we visually inspected residue-residue contacts in PyMOL (The PyMOL Molecular Graphics System, Version 3.0, Schrödinger, LLC, 2024) and analyzed key binding residues within the C-terminal region of AMG386, which contains previously identified conserved sequence motifs. Specific interactions were identified based on proximity thresholds (e.g., < 3.5 Å for hydrogen bonds) and π-stacking geometries, supporting the structural reliability of the predicted complex.

## Cell proliferation

HUVEC (2–4 × 10^3^) were seeded in propagation medium on gelatin (0.25%) pre-coated 96-well plates. After overnight incubation, culture medium was changed to starvation medium only or with FGF1 or/and Ang2 for 48 h at the indicated concentrations. ^3^H-thymidine (PerkinElmer, No. NET027WW001MC) was added (0.5 µCi) to the wells for 24 h. Plates were frozen at −80 ºC and after thawing cells were transferred to glass fiber filters (PerkinElmer, No. 1450 − 421). Incorporated radioactivity was counted in a liquid scintillation counter (PerkinElmer, No. MicroBeta-2450).

### Endothelial cell migration in monolayer culture; scratch assay

HUVEC were seeded (8 × 10^4^ cells) in 24-well plates pre-coated with gelatin (0.25%) in complete endothelial cell culture medium. After 6 h incubation, culture medium was changed to starvation medium and cultures incubated overnight. A scratch was made in the confluent HUVEC monolayer using a 10 µl tip. Fresh starvation medium containing FGF1 or/and Ang2 and/or AMG386 was added to the cells and cultures were incubated for 12–16 h at 37 ºC. Cultures were imaged using the Olympus IX51 Inverted Fluorescence (UplanFL) microscope with a 4x objective lens; images were acquired with cellSens Imaging Software (Olympus). For each scratch, 3 images were acquired, covering the left, middle and right regions, encompassing 85% of the entire scratch (the remaining part of the scratch, at the outer margins, could not be accurately imaged). This ensured unbiased capture of the scratch to reflect regional variation. Images obtained at time 0 (when the scratch reflects the starting maximum size) served as reference for calculation of degree of closure at subsequent time-points. We selected the 12- and 16-hour time-points to reflect the onset of cell movement after the scratch is applied and the 16 h timepoint to assess the progress of wound closure. The images were analyzed by ImageJ software (NIH) with the wound healing size tool plugin [30]. The results are presented as % wound closure of the wound size at time 0.

## Endothelial cell transwell migration assay

HUVEC (5 × 10^5^) were seeded on the upper chamber of HTS Transwell 24-well plates with 8.0 μm pore size PET membrane (cell growth area 0.3 cm^2^, Falcon No. 353097) in starvation medium (0.3 ml) for 2 hours. The bottom chamber was filled with 200 µl endothelial cell starvation medium. Ang2 was then added to some of the upper chambers (100 ng/ml for 30 min). After 30 min, the top chamber was transferred to a new bottom chamber prefilled with starvation medium (200 µl) supplemented with 50 ng/ml FGF1 where indicated. After 4 h incubation, the upper membrane was wiped clean using a cotton swab. Migrated cells on the lower surface of the membrane separating the upper and lower chambers were fixed with 4% formaldehyde for 20 min and stained with 0.5% crystal violet for one hour. After washing in water, the membrane was imaged using an Olympus IX51 Inverted Fluorescence (UplanFL) microscope, at 4× and 20× magnification and photographed (3–4 images/membrane). The number of cells migrated was counted in 3 or 4 images acquired at 20×magnification (representing ~ 1.0% of the total membrane surface, 300 mm^2^), and the results were averaged. The average cell count/image was then normalized to the total membrane area.

## Mouse wound healing assay

The assay was performed essentially as described [31]. Seven- to nine-weeks-old C57BL/6 mice were anaesthetized with Isoflurane. Two wounds were applied using a 5 mm biopsy punch (Acuderm, No. P525) on the upper back on each side. A silicone splint ring (ThermoFisher, No. P18178) was attached and secured with 6 stiches. The wounds were covered with a gelatin sponge (5 mm in diameter to fit into the wound created by the punch biopsy) (Vetspon FLEX, Elanco No. 96001) and occlusive dressing (Opsite Flexifix, Smith & Nephew, No. 66000040). The gelatin sponge was changed at each time-point the wound was opened, usually every other day. Overall, 41 mice (81 wounds) were used in the wound-healing experiments presented. Wound closure in untreated (control) wounds was evaluated in 42 separate wounds; in mice treated topically with FGF2, 12 wounds; in mice treated with the combination of FGF2 and Ang2, 3 wounds; in mice treated with topical Ang2, 6 wounds; in mice treated with topical AMG386, 8 wounds; and in mice treated systemically with REGN910/Nesvacumab, 10 wounds. Ang2 (1 µg), FGF2 (Trafermin, Creative Biomart, No. THP-0292, 1 µg), and AMG386 (45 µg) diluted in PBS were applied to the wounds once/day or every other day. REGN910/Nesvacumab (ProteoGenix, No. PX-TA1311) was injected subcutaneously (s.c.; 2.5 mg/kg in 50 µl PBS) twice/week beginning on the day of wounding. Wounds were photographed at the time of treatment. The wound area was measured with ImageJ using the inner splint hole as reference for area calculations.

### Tissue fixation, Immunofluorescence staining, imaging and image quantification

Skin fragments harvested on day 4 after wounding were pre-fixed in 4% PFA for 24 h at 4 ºC, soaked into 17% sucrose in PBS for 24 h at 4 ºC, and embedded in OCT (Tissue-Tek OCT compound, Sakura, No. 4583). For immunostaining, 8 μm tissue sections were incubated in Uni-Trieve solution (Innovex Biosciences, No. NB325) overnight at 45 ºC. After washing with 1% Triton X-100 in PBS, sections were incubated in blocking solution (10% glycerol, 0.4% Triton X-100, 0.5% BSA, 1X Tris-buffered saline/TBS), as described [32] for 1 h at room temperature. Skin sections were incubated overnight at 4 ºC with rat anti-CD31 antibody (1:100 dilution, BD Biosciences, No. 550274), rabbit anti-SMA antibody (1:50 dilution, Cell Signaling Technology, No. 19245), rat anti-CD45 antibody (1:50 dilution, BioLegend, No. 103101), rabbit anti-K14 antibody (1:100 dilution, BioLegend, No. 905304), and rabbit anti-pFGFR antibody (1:100 dilution, Cell Signaling Technology, No. 3471). After washing, the sections were incubated for one hour at 4 ºC with Alexa Fluor (AF) 647 labeled donkey anti-rabbit IgG (1:100 dilution, Invitrogen, No. A31573), AF488 donkey anti-rat IgG (1:100 dilution, Invitrogen, No. A21208), AF488 donkey anti-rabbit IgG (1:100 dilution, Invitrogen, No. A21206) or AF594 donkey anti-rat IgG (1:100 dilution, Invitrogen, No. A21209). The slides were washed in Tris-buffered saline (TBS) supplemented with 0.4% Triton X-100, 0.5% bovine serum albumin (BSA) and 10% glycerol/, re-fixed with 4% PFA for 20 min at room temperature and mounted with DAPI-containing mounting media (Sigma, No. F6057). For the quantification of fluorescent cell populations in wounds, tissue sections of whole skin wounds were imaged through the confocal microscopes Stellaris 8 FALCON FLIM, Stellaris 5 STED (Leica Microsystems) or LSM 880 Airyscan (Zeiss), and the acquired images were exported as separate TIFF files using the LAS X software (Life Science Microscope software; Leica Microsystems) or ZEN software (Zeiss). Regions of interest (ROIs) were generated from DAPI-stained TIFF images using the software CellPose [33] on Biowulf (the high-performance computing system at NIH). Overlapping DAPI-stained nuclei were separated by the watershed method in ImageJ on Biowulf. Fluorescent images of CD31^+^, CD45^+^, SMA^+^ nucleated (DAPI^+^) cells were converted to binarized images using the “Auto Threshold” function of ImageJ in Biowulf. A custom Python script (Counting_CD31_positive_cells_with_pericyte_coverage.py deposited in GitHub, URL https://github.com/HidetakaOhnuki-NCI/Wound-healing-analysis/blob/main/Counting_CD31_positive_cells_with_pericyte_coverage.pyessels.py) was used to quantify CD31^+^ cells with adjacent (1 μm range) SMA^+^ cells in the wounds. For counting CD31^+^ cells, CD45^+^ cells and myofibroblasts, Python scripts “Counting_CD31_positive_cells.py”, “Counting_CD45_positive_cells.py” and “Counting_myofibroblasts.py” were used (all scripts were deposited in GitHub, https://github.com/HidetakaOhnuki-NCI/Wound-healing-analysis/tree/main). CSV files generated by the Python scripts contain X and Y axis values, indicating individual cell positions of CD31^+^ cells with/without pericyte-coverage, CD45^+^ cells and myofibroblasts (αSMA^+^ cells without CD31^+^ cell contact) in the tissue image. The results of fluorescence immunostaining (calculated in Excel) are expressed as number or percentage of cells in wound sections ranging 0–200, 201–400, 401–600, 601–1200, 1201–1400,1401–1600, and 1601–1800 μm ranges from the wound center. The position of the wound center was determined by CK14 staining as the middle position of the open wound.

### Statistical analysis

Statistical tests, significance levels, and sample sizes are detailed in each figure legend. Statistical analyses were performed using GraphPad Prism 10 software. The data are reported as the mean ± standard deviation (SD) or standard error of the mean (SEM). We have applied the Shapiro-Wilk test to evaluate if the results are normally distributed. Statistical comparisons between two groups were performed by the parametric paired or unpaired two-sided Student’s *t*-test. Differences among three or more groups were assessed by analysis of variance with the parametric 2-way ANOVA for multiple comparisons using Sidak’s correction. *P* values < 0.05 were considered statistically significant.

## Results

### FGFR2 and Ang2 are proximal to each other within endothelial cells

**Fig. 1:**
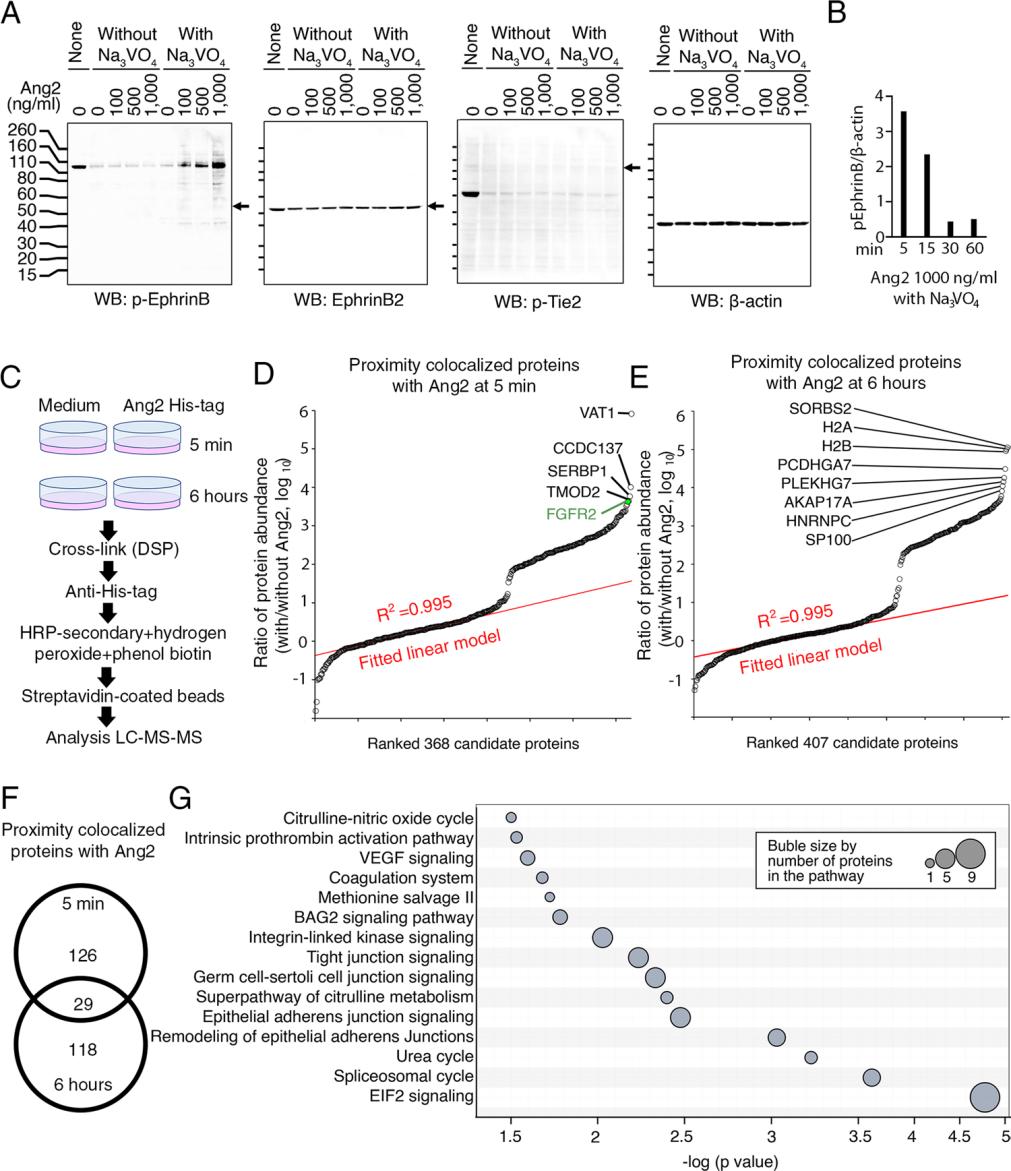
Ang2 activates a previously unidentified protein in endothelial cells. **A **HUVEC were incubated for 5 minutes with Ang2 (100, 500 and 1000 ng/ml with or without sodium orthovanadate (Na_3_VO_4_) after overnight starvation. None: HUVEC cultured under standard conditions. Cell lysates were tested for p-EphrinB^T324/329^, EphrinB2, p-TIE2, and ß-actin. After 5 minutes incubation with Ang2, a ~130kDa band is recognized by p-EphrinB but not by EphrinB2 or p-TIE2 antibodies. **B** Time dependent decrease in intensity of the ~130kDa band identified by p-EphrinB antibodies. Relative band intensity was measured by ImageJ. **C **Experimental design for proximity labeling. Starved HUVEC were incubated for 5 minutes or 6 hours with or without His-tag Ang2 (100 ng/ml). After crosslinking (2 mM DSP), incubation with the primary anti-His-tag antibody, addition of HRP-conjugated secondary antibody plus hydrogen peroxide and phenol biotin, protein purification with streptavidin-coated Dynabeads, the eluted proteins were analyzed by LC-MS-MS. **D** Relative abundance of selected proteins identified by LC-MS-MS in HUVEC incubated for 5 minutes with medium only or with Ang2. FGFR2 is identified among the most abundant proteins specifically associated with Ang2 in HUVEC by proximity labeling. The results are evaluated by model-based analysis of proteomic data (MAP). **E **Relative abundance of selected Ang2-associated proteins detected by proximity labeling in HUVEC incubated for 6 hours with Ang2. **F **Venn diagram depicts the number of Ang2-interacting proteins unique to or common to the 5 minutes and 6 hours incubation. **G **Pathway enrichment analysis applied to the 368 Ang2-proximal proteins identified in HUVEC after 5 minutes incubation with Ang2

Previously, we showed that Ang2 induces Tie2 tyrosine receptor phosphorylation (Y992; AF2720) in primary human umbilical vascular endothelial cells (HUVEC) with delayed kinetics compared to other ligands activating their respective tyrosine receptors in endothelial cells; Tie2 activation was detected after 6 h, but not at earlier time-points (15–120 min) [32]. This delayed Ang2-induced activation of Tie2 is consistent with other observations [34]. We now found that Ang2 (100–1000 ng/ml) also induces the early (5 min post exposure) appearance of a band at ~ 130 kDa in the presence of the phosphatase inhibitor sodium orthovanadate (Na_3_VO_4_) (Fig. 1A). This band is recognized by a p-EphrinB^T324/329^ antibody that binds Ephrin B1, B2, and B3 but is not recognized by antibodies to phospho (p)-Tie2^Y992^ or antibodies to EphrinB2 (Fig. 1A) [32]. Prominently observed at 5 min in HUVEC, this ~ 130 kDa band markedly decreases in intensity by 30 and 60 min, suggestive of rapid activation and deactivation (Fig. 1B). It is unlikely that this ~ 130 kDa band reflects B-type Ephrins because Ephrins are smaller proteins (~ 55–60 kDa) and because an antibody to total EphrinB2 (ab150411), the most abundant B-type Ephrin in HUVEC [35], does not recognize this ~ 130 kDa band while recognizing the expected ~ 55 kDa EphrinB2-related molecule (Fig. 1A).

To identify the protein associated with this ~ 130 kDa band that is rapidly and transiently induced by Ang2, we used a proximity-based labeling method to guide biotin deposition onto proteins proximal to Ang2 followed by affinity purification of these proteins and analysis by liquid chromatography with tandem mass spectrometry (LC-MS-MS) [36, 37] (schematic of the experiment, Fig. 1C). Based on its kinetic detection, showing that the ~ 130 kDa band is most intensely induced by Ang2 at 5 min and is no longer or minimally detected after 1 h (Fig. 1B) [32], HUVEC were stimulated for 5–6 h with His-tag Ang2 or medium only. LC-MS-MS identified 368 candidate proteins colocalized with Ang2 at the 5 min time-point (Fig. 1D) and 407 at the 6-hour time-point (Fig. 1E). After ranking the candidate proteins based on their abundance, we found that FGFR2 was one of the most abundant proteins detected at 5 min (Fig. 1D) but not detected at 6 h (Fig. 1E), when Ang2 no longer induces the ~ 130 kDa band (Fig. 1B). After applying a model-based analysis of proteomic (MAP) data to detect proteins with a significant change in abundance [38], we found that only 126 Ang2-proximal proteins were uniquely present at 5 min (from the original 368 candidates) (Suppl. Table 1), and 118 Ang2-proximal proteins were present at 6 h (from the original 407 candidates) (Suppl. Table 2). Overall, 29 proteins were detected at both time points (Fig. 1F). The Ang2-proximal proteins detected uniquely at 5 min were enriched with proteins defining signaling from the eukaryotic initiation factor 2 (EIF2), and to a lower degree with proteins defining signaling from tight junctions, as well as integrin-linked kinase signaling (Fig. 1G). Taken together, these results suggested that FGFR2 is adjacent to Ang2 in HUVEC, raising the possibility that Ang2 may bind to FGFR2.

### Ang2 directly binds and activates FGFR2

**Fig. 2:**
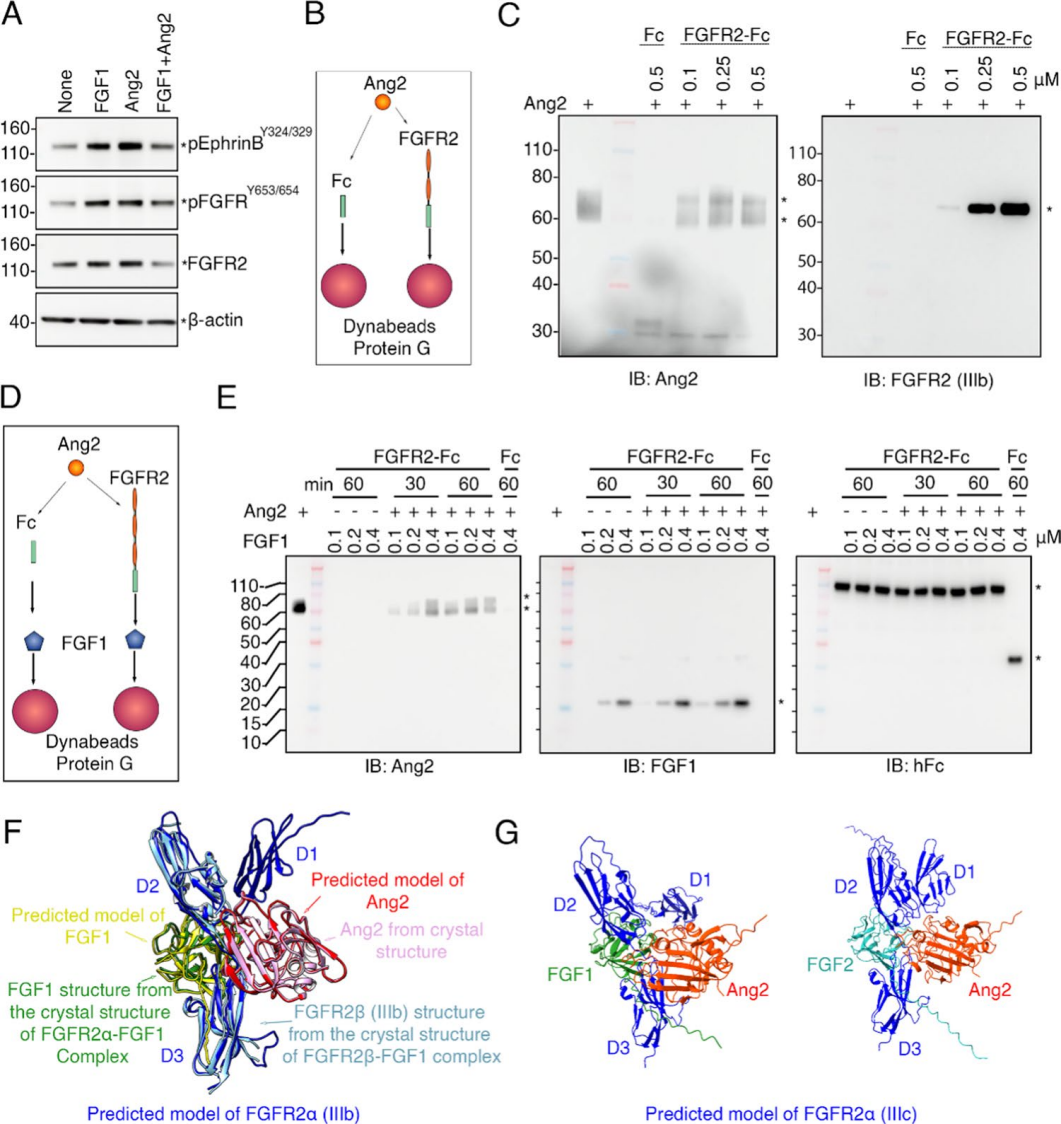
Recombinant Ang2 binds to recombinant FGFR2-Fc. **A **Lysates prepared from HUVEC in starvation medium for 1 hour and then incubated with FGF1 (5 nM, 5 min) or/and Ang2 (5 nM, 5 min) in the same medium were analyzed by Western blotting; antibodies to p-FGFR^Y653/654^and FGFR2 recognize a band at ~130 kDa. Representative of 3 experiments. **B **Schematic of the pull-down experiment. FGFR2: FGFR2ß (IIIb)-Fc. **C **Ang2 (0.1 μM) specifically binds to recombinant FGFR2ß (IIIb)-Fc (0.1-0.5 μM) but not to human IgG-Fc (Fc, 0.5 μM). The precipitated proteins were immunoblotted with antibodies to Ang2 (left) or to FGFR2ß (IIIb) (right). The asterisks point to bands specifically identifying Ang2 (left) and FGFR2 (right). Representative of 3 experiments. **D **Schematic of the pull-down experiment. FGFR2: FGFR2α (IIIc)-Fc. **E** Ang2 (0.1 μM) does not compete with the binding of FGF1 (0.1-0.4 μM) to FGFR2α (IIIc)-Fc (0.2 μM). IgG-Fc used at 0.2 mM. The precipitates were immunoblotted with antibodies to Ang2, FGF1, or anti-human Fc (hFc). The asterisks point to Ang2 (left), FGF1 (middle), and to FGFR2-Fc or Fc (right). Representative of 3 experiments. **F** Structural modeling of the Ang2, FGF1 and FGFR2α (IIIb) trimeric complex from amino acid sequences by AlphaFold2-Multimer. Red: predicted structure of Ang2 in the predicted trimeric complex. Pink: Ang2 structure from the crystal structure of Ang2 alone (PDB 1z3s). Blue: predicted structure of FGFR2α (IIIb). Light blue: FGFR2ß (IIIb) structure from the crystal structure of FGFR2ß (IIIb)-FGF1 dimeric complex (PDB 1djs). Yellow: predicted structure of FGF1. Green: FGF1 structure from the crystal structure of FGFR2ß (IIIb)-FGF1 complex (PDB 1djs). The three predicted protein structures are largely consistent when superimposed on the respective crystal structures. The predicted structure suggests that Ang2 and FGF1 interact with FGFR2 at distinct sites. **G** Structural models of the Ang2, FGF1 and FGFR2α (IIIc) trimeric complex (left) and the Ang2, FGF2 and FGFR2α (IIIc) trimeric complex (right) from amino acid sequences by AlphaFold3

Based on the results from proximity labeling, we examined whether the ~ 130 kDa band, detected by p-EphrinB antibodies in lysates of HUVEC induced by Ang2, corresponds to FGFR2. By immunoblotting, antibodies to p-FGFR^Y653/654^, which detect Y653/654 of FGFR-1, −2, −3 and − 4, recognized a band at ~ 130 kDa in lysates of HUVEC incubated with FGF1 (a ligand for FGFR2), Ang2, and FGF1 + Ang2 (Fig. 2A). The same band was also recognized by specific antibodies to FGFR2 indicating that this band likely detects FGFR2. These results suggested that Ang2 may bind FGFR2.

To test Ang2 binding to FGFR2, we incubated Ang2 with recombinant FGFR2ß (IIIb)-Fc or control IgG-Fc followed by precipitation with protein-G coated Dynabeads and immunoblotting (Fig. 2B, experiment schematic). The precipitates from Ang2 + FGFR2ß (IIIb)-Fc contained Ang2 and FGFR2 whereas precipitates from Ang2 + IgG-Fc did not. As shown, Ang2 antibodies recognized the presence of Ang2 in the precipitates from Ang2 + FGFR2ß (IIIb)-Fc but not from Ang2 + IgG-Fc (Fig. 2C, left). Also, FGFR2ß (IIIb) antibodies recognized FGFR2 in the precipitates from Ang2 + FGFR2ß (IIIb)-Fc but not from Ang2 + IgG-Fc (Fig. 2C, right). These experiments showed that Ang2 can specifically bind to FGFR2ß (IIIb).

FGFR2 has two isoforms, the IIIb type and the IIIc type, that differ in the carboxyl half of the third extracellular Ig domain due to alternative splicing; these isoforms are preferentially expressed in different cell types, and while they respond to a somewhat different spectrum of FGFs, both types are high-affinity receptors for FGF1 [41, 42]. We examined whether Ang2 can bind to FGFR2ß (IIIb) and FGFR2α (IIIc) and found that Ang2 can bind to FGFR2ß (IIIb) and FGFR2α (IIIc) (Suppl. Figure 1A).

Next, we tested whether Ang2 can compete with FGF1 for binding to FGFR2α (IIIc). To this end, we pre-incubated Ang2 (0.1 µM) with FGFR2α (IIIc)-Fc for 30–60 min (4 °C) along with control IgG-Fc. Subsequently, FGF1 (0.1–0.4 μM) was added to Ang2-FGFR2α (IIIc)-Fc, and to IgG-Fc, and incubation continued for 1 h (4 °C) (Fig. 2D schematic of the experiment). The precipitates (from protein-G coated Dynabeads) were evaluated for the presence of Ang2 (Fig. 2E left), FGF1 (Fig. 2E middle) and FGFR2-Fc (Fig. 2E right). The immunoblotting results show that recovery of FGF1 was not reduced by Ang2 preincubation with FGFR2α (IIIc)-Fc. Rather, the recovery of FGF1 was somewhat time-dependently increased by Ang2 pre-incubation with FGFR2α (IIIc)-Fc (Fig. 2E middle). The results additionally show that the recovery of Ang2 was also somewhat increased by addition of FGF1 after 30 min preincubation of Ang2 with FGFR2α (IIIc)-Fc (Fig. 2E left). These results confirm that Ang2 specifically binds to FGFR2 and suggest that Ang2 and FGF1 can facilitate each other’s binding to FGFR2.

To understand how Ang2 may bind to FGFR2, we undertook several structural predictions with AlphaFold2-Multimer [27] (10.1101/2021.10.04.463034). Structures of the Ang2-FGFR2α (IIIb)-FGF1 complex were modeled from amino acid sequences of human Ang2 (residues 281–494), FGFR2α (IIIb) (residues 1–362) and FGF1 (residues 1–155). The best complex of Ang2 with FGF1 and FGFR2α (IIIb) with the highest Predicted Template Modeling (pTM: iptm + ptm for multimer) score of 0.55 is shown in Fig. 2F. This model is consistent with the generally high estimation of 3-dimensional accuracy shown by the matrix of Predicted Aligned Error (PAE) scores (Suppl. Figure 1B), and the fact that AlphaFold2 was trained on these structures. As seen in the predicted structure, Ang2 binds in-between and interacts with the two immunoglobulin-like domains (/D2/Ig2 and/D3/Ig3) of FGFR2α (IIIb), and FGF1 binds to the opposite side of the immunoglobulin domains (/D2/Ig2 and/D3/Ig3) of FGFR2. The face of Ang2 interacting in the complex with FGFR2α (IIIb) is the same as in the crystal structure of Ang2 interacting with Tie2 (PBD ID: 2gy7). Thus, structural simulations suggest that Ang2 similarly binds to FGFR2α (IIIc) and to Tie2, and that the location of Ang2 binding to FGFR2 differs from that of FGF1. Additional structural simulations with AlphaFold3 [29] (10.1038/s41586-024-07487-w (2024)) examined the binding of Ang2 to FGFR2α (IIIc), since FGF2 is a selective ligand for FGFR2α (IIIc), unlike FGF1 that binds to all FGFRs [41, 42]. The best trimeric complexes of Ang2, FGFR2α (IIIc), FGF1 and Ang2, FGFR2α (IIIc), FGF2 (Fig. 2G) show a generally high estimation of 3-dimensional accuracy by the matrix of Predicted Aligned Error (PAE) scores (Suppl. Figure 1C and D).

**Fig. 3:**
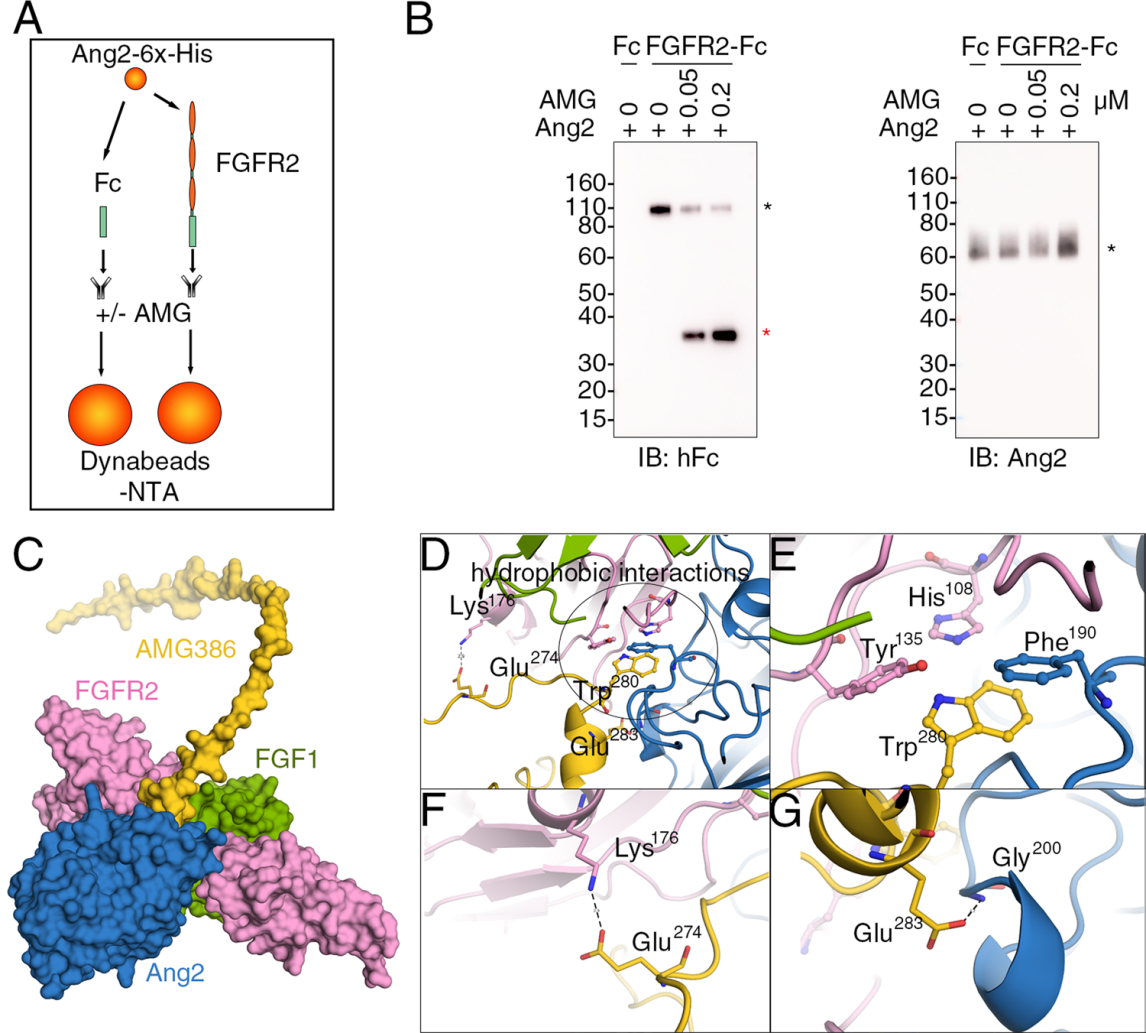
The Ang2 inhibitor AMG386 reduces Ang2 binding to FGFR2-Fc. **A** Schematic of the pull-down experiment. FGFR2α (IIIc)-Fc. **B** AMG386 (AMG, 0.2 or 0.05 μM) inhibits the binding of Ang2 (0.1 μM) to FGFR2-Fc (0.2 μM). IgG-Fc (Fc, 0.2 μM). The precipitates were immunoblotted with antibodies to Fc and Ang2. The black asterisk points to FGFR2-Fc (left) and Ang2 (right); the red asterisk points to AMG386. Representative experiment (of three performed). **C **Overall complex structure of AMG386 (yellow) bound to Ang2 (blue), FGF1 (green), and FGFR2α (IIIb) (pink) is shown as a surface representation. AMG386 occupies the main binding cavity of the complex, highlighting its central role in coordinating interactions with the other proteins. **D** and** E** Trp^280^ from AMG386 (yellow) forms hydrophobic interactions with nearby residues, including Tyr^135^ and His^108^ from FGFR2 (pink) and Phe^190^ from Ang2 (blue). These interactions include π-stacking between Trp^280^ (AMG386) and Phe^190^ (Ang2), contributing to the stabilization of the complex. Additional π-π stacking interactions involving His^108^ (FGFR2) and Phe^190^ (Ang2) underline the importance of aromatic side chains in maintaining the structural integrity of the complex. **F** A hydrogen bond between Lys^176^ (FGFR2, pink) and Glu^274^ (AMG386, yellow) reinforces the binding interface.** G** Another hydrogen bond is observed between Glu^283^ (AMG386, yellow) and Gly^200^ (Ang2, blue), further enhancing the specificity and stability of the interaction

Based on these structural simulations, we tested if an inhibitor of Ang2 would reduce Ang2 interaction with FGFR2. We selected AMG386, a peptide (14 amino-acids)-Fc fusion protein (peptibody) that inhibits Ang1 and Ang2 binding to Tie2 [44–46]. We incubated (60 min, 4 °C) His-tagged recombinant Ang2 with recombinant FGFR2α (IIIc)-Fc or control IgG-Fc with or without AMG386, followed by precipitation with NTA-Dynabeads (for purification of His-tagged fusion proteins) and immunoblotting the precipitates (Fig. 3A, experiment schematic). The results (Fig. 3B, left) show that the recovery of FGFR2 was reduced when AMG386 was present during incubation of FGFR2-Fc and Ang2, whereas the recovery of Ang2 was not reduced (Fig. 3B, right). This indicates that AMG386 specifically inhibits the binding of Ang2 to FGFR2α (IIIc) suggesting AMG386 interaction within the complex of FGFR2, FGF1 and Ang2.

We utilized AlphaFold3 [29] (10.1038/s41586-024-07487-w (2024)) to obtain a detailed view of predicted molecular interactions within a complex of AMG386 with Ang2, FGF1 and FGFR2. The best complex of AMG386, Ang2, FGF1 and FGFR2α is shown in Fig. 3C and related Suppl. Figure 1E and F. Our analysis reveals that AMG386 binding involves a combination of hydrophobic interactions, π-stacking, and hydrogen bonds with Ang2 and FGFR2. Key residues, such as Trp^280^ in AMG386, engage in hydrophobic and π-π stacking interactions with aromatic residues from Ang2 (Phe^190^) and FGFR2 (His^108^) (Fig. 3D, E), while hydrogen bonds further stabilize the interface, notably between AMG386-Glu^274^ and FGFR2-Lys^176^, as well as AMG386-Glu^283^ and Ang2-Gly^200^ (Fig. 3F, G). Notably, AMG386 is not predicted to interact with FGF1. These interactions collectively underscore the predicted molecular basis of AMG386 binding affinity and specificity within the complex.

### Ang2 inhibits FGF1 signaling

**Fig. 4:**
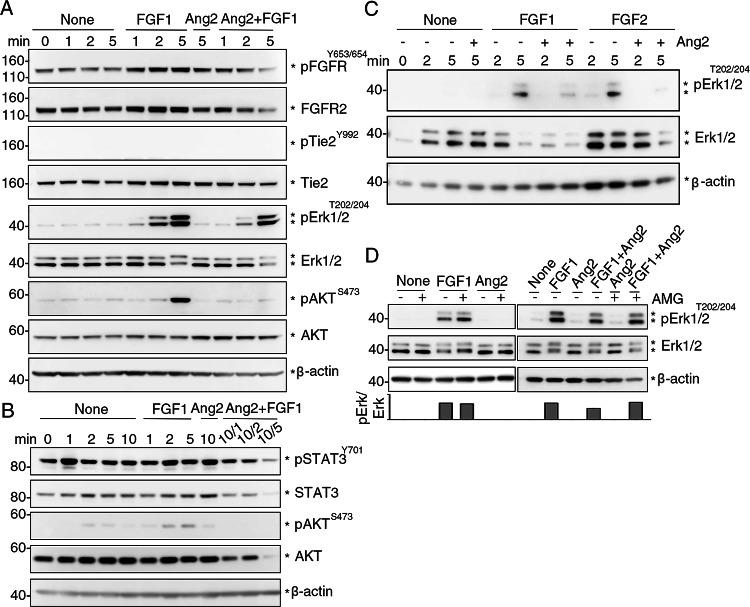
Ang2 reduces FGF1-induced activation of FGFR, Erk1/2, STAT3 and AKT in HUVEC**. A **HUVEC were incubated in starvation medium only (None), with FGF1 (3 nM), with Ang2 (3 nM) or with Ang2 (3 nM) + FGF1 (3 nM) for the indicated times (min). Cell lysates were immunoblotted with the indicated antibodies. When FGF1 + Ang2 were added to the cells, the incubation time of Ang2 was 5 min and the incubation time of FGF1 was either 1, 2 or 5 minutes (displayed as 5/1, 5/2, 5/5). Representative results of 3 experiments. **B** HUVEC were incubated in starvation medium only (None), with FGF1 (3 nM), with Ang2 (3 nM) or with Ang2 (3 nM) + FGF1 (3 nM) for the indicated times (min). When Ang2 + FGF1 were added to the cells, the incubation time of Ang2 was 10 min and the incubation time of FGF1 was either 1, 2 or 5 minutes (displayed as 10/1, 10/2, 10/5). Representative of 3 experiments.** C** HUVEC were incubated in starvation medium only (None), with FGF1 (3 nM), FGF2 (3 nM), Ang2 (3 nM), Ang2 (3 nM) + FGF1 (3 nM) or with Ang2 (3 nM) + FGF2 (3 nM). When Ang2+FGF1 or Ang2+FGF2 were added together to the cells, the incubation time was either 2- or 5-min. Representative of 3 experiments.** D **Effects of AMG386 on p-Erk1/2 levels in HUVEC activated by FGF1 alone, Ang2 alone, or FGF1 + Ang2. HUVEC were cultured in starvation medium only (none), FGF1 only (5 nM; 5 min), Ang2 only (5 nM; 10 min) or FGF1 (5 nM; 5 min) + Ang2 (5 nM; 10 min). Where indicated, AMG386 (AMG, 315 nM) was added to HUVEC 60 min prior to the addition of FGF1 alone, Ang2 alone or Ang2 + FGF1. Relative band intensity (p-Erk/total Erk) is shown in the bar graph. Representative of 3 experiments

Next, we examined potential signaling effects of Ang2 binding to FGFR2 in HUVEC and compared to those of FGF1 binding to FGFR2. Individually, Ang2 and FGF1 (3 nM) induced the phosphorylation of FGFR^Y653/654^ at 5 min in HUVEC (Fig. 4A), without inducing Tie2 phosphorylation (Fig. 4A and Suppl. Figure 2 A). The failure of Ang2 to induce Tie2 phosphorylation in Tie2^+^ HUVEC at these time-points is consistent with the delayed kinetics of Tie2 phosphorylation in these cells [32]. When present together, Ang2 (5 min) and FGF1 (1, 2–5 min), reduced the levels of FGFR^Y653/654^ phosphorylation induced by either Ang2 or FGF1 alone at 5 min (Fig. 4A). Furthermore, Ang2 and FGF1 together reduced the levels of FGFR2 protein (Fig. 4A), consistent with activated FGFRs undergoing casitas B lineage lymphoma (CBL)-induced ubiquitylation [46]. Unlike FGF1, Ang2 minimally promoted Erk1/2^T202/204^, AKT^S473^ or STAT3^Y701^activation (Fig. 4A, B, and Suppl. Figure 2 A). However, Ang2 inhibited the FGF1-induced phosphorylation of Erk1/2^T202/204^, AKT^S473^ and STAT3^Y701^, and reduced protein levels of total Erk1/2, AKT and STAT3 (Fig. 4A, B). This reduction of total protein levels is consistent with previous results showing that degradation is an important mechanism for regulation of active protein kinases [47]. In additional experiments we tested the effects of Ang2 on FGF2-induced signaling in HUVEC. Like the results with FGF1, Ang2 inhibited FGF2-induced Erk1/2 activation in HUVEC (Fig. 4C).

In other experiments, we tested if the Ang2 inhibitor AMG386 [44–46] alleviates Ang2 inhibition of FGF1-induced signaling. In HUVEC (Fig. 4D), we found that AMG386 minimally affects FGF1-induced Erk phosphorylation (left) but mitigates Ang2 inhibition of FGF1-induced Erk1/2 activation (right). These observations support a role of Ang2 as an inhibitor of FGF1-induced Erk1/2 signaling.

**Fig. 5:**
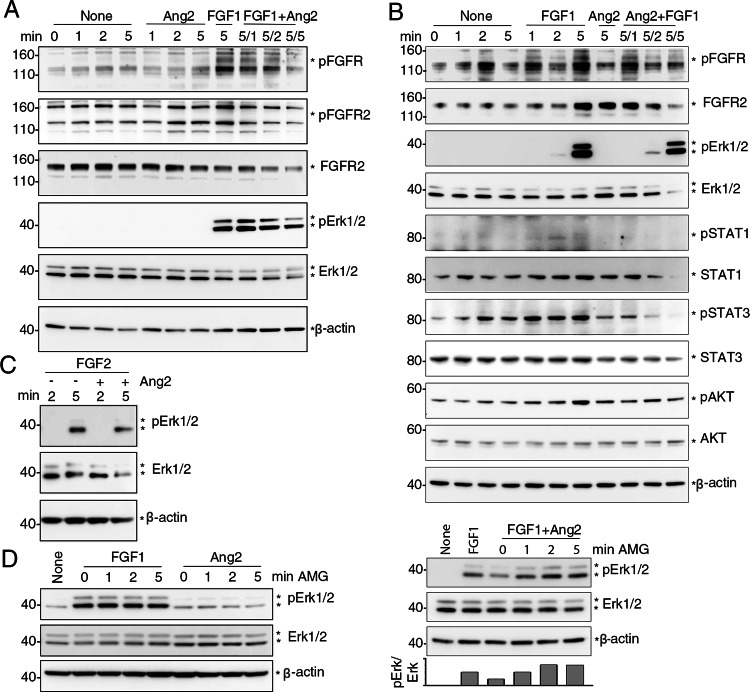
Ang2 reduces p-FGFR, p-Erk1/2, p-STAT3 and p-AKT in HEK293T cells.** A, B** HEK293T cells were incubated in starvation medium only (None), with Ang2 (3 nM), with FGF1 (3 nM), or with Ang2 (3 nM) + FGF1 (3 nM) for the indicated times (min). Cell lysates were immunoblotted with the indicated antibodies. Representative results from 3 experiments. When FGF1 + Ang2 were added to the cells, the incubation time of FGF1 was 5 min and the incubation time of Ang2 is either 1, 2 or 5 minutes (displayed as 5/1, 5/2, 5/5). **C** HEK293T cells were incubated in starvation medium with FGF2 (3 nM) alone (2 or 5 min), or with Ang2 (3 nM), 5 min. The results show Ang2 + FGF2 for 5 min reduce FGF2-induced Erk1/2 activity (5 min). **D **Left panel: HEK293T cells were incubated in starvation medium only (None), FGF1 only (5 nM; 5 min), or with Ang2 (5 nM; 5 min) without or with AMG386 (AMG, 5 ng/ml; 1, 2, or 5 min). Right panel: HEK293T cells were incubated with medium only (None), FGF1 only (5 nM; 5 min), or FGF1 (5 nM; 5 min) + Ang2 (5 nM; 5 min), with or without AMG386 (5 ng/ml; 1, 2, or 5 min). Relative band intensity (p-Erk/total Erk) is shown in the bar graph. Representative of 3 experiments

We then examined whether the presence of Tie2 was required for Ang2-induced activation of and signaling by FGFRs. Therefore, we utilized the human HEK293T cells, which do not express *TEK*, the gene coding for Tie2; HEK293 cells express *FGFR-1*, *−2*, *−3* and *− 4*, albeit to a lower overall level compared to HUVEC (Suppl. Figure 3A-C). In HEK293T cells, FGF1 (3 nM) induced FGFR2^Y653/654^ activation whereas Ang2 (3 nM) did not or minimally (Fig. 5A, B). However, Ang2 time-dependently (1–5 min) reduced FGFR2^Y653/654^ phosphorylation induced by FGF1 and protein levels of FGFR2. Additionally, Ang2 time-dependently reduced Erk1/2 phosphorylation^T202/204^ induced by FGF1, associated with a reduction of total Erk1/2 protein; Ang2 alone did not activate Erk1/2 phosphorylation^T202/204^ (Fig. 5A, B). Similarly, Ang2 did not activate STAT3^Y701^ or AKT^S473^ but reduced p-STAT3^Y701^ and p-AKT^473^ induced by FGF1 (Fig. 5B). In addition to inhibiting FGF1 signaling, Ang2 also inhibited FGF2-induced Erk1/2 activation in HEK293T cells (Fig. 5C).

We tested the effects of the Ang2 inhibitor, AMG386, in HEK293T cells. The results show that AMG386 has no effect on FGF1-induced Erk activation (Fig. 5D, top panel) but mitigates the inhibitory effects of Ang2 in HEK293T cells, as it rescued levels of FGF1-induced p-Erk1/2 (Fig. 5D, bottom panel). Overall, these results provide evidence that Ang2 functions as an inhibitor of FGF1- and FGF2-induced FGFR activation and downstream signaling in cells that do or do not express Tie2.

### Ang2 regulates endothelial cell motility induced by FGF1

**Fig. 6:**
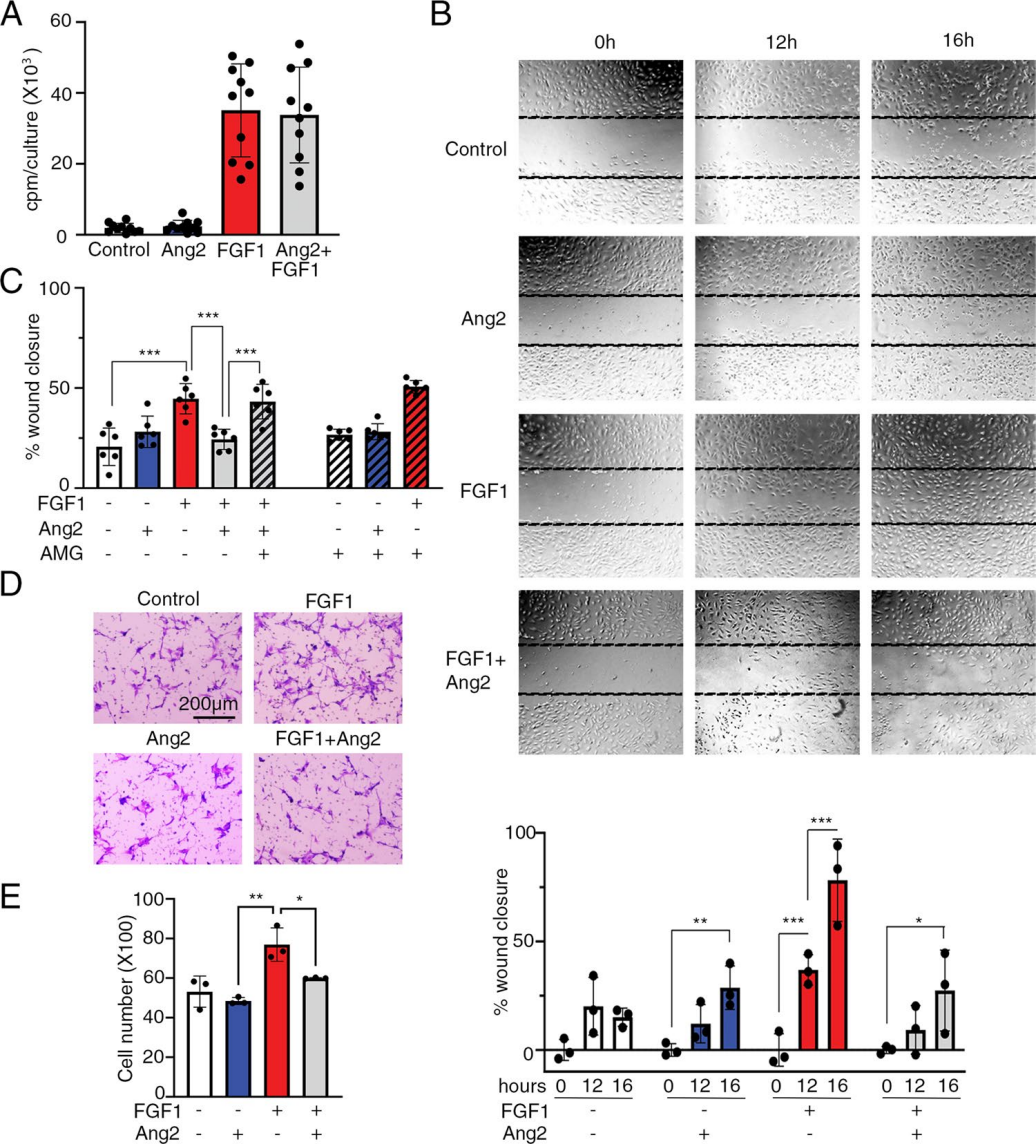
Ang2 impairs endothelial cell migration induced by FGF1. **A** Effects of Ang2 (100 ng/ml) and FGF1 (3 ng/ml) individually or together on HUVEC proliferation after 72 hours incubation. Results from ^3^H thymidine incorporation are expressed as cpm/culture. Dots reflects results of individual experiments performed in triplicate cultures; experimental means (±SD) are reflected by the bar graphs and error bars. **B** Ang2 reduces FGF1-induced wound healing in vitro. Images from a representative wound healing assay (of 5 assays) evaluated at 0, 12 and 16 hours (h) after HUVEC wounding. Ang2 (100 ng/ml), FGF1 (10 ng/ml) were added individually or together to the wounded HUVEC monolayers. Quantification of the results from triplicate cultures. The results of % wound closure from individual values (shown as dots) are expressed as mean (±SD), reflected by the error bars. Representative of 5 experiments. **C **The Ang2 inhibitor, AMG386 (AMG, 5 ng/ml) mitigates inhibition of wound healing by Ang2 (100 ng/ml) in the presence of FGF1 (10 ng/ml). Results of HUVEC wound closure from 4 experiments (evaluated at 12 hours after wounding) are presented as individual dots and means (±SD), reflected by the error bars. **D, E** Ang2 reduces FGF1-induced HUVEC transmigration. HUVEC (5x10^5^) were tested in transmigration assays using Transwells (8.0 µm pore size) with or without Ang2 (100 ng/ml) and FGF1 (50 ng/ml). The number of cells migrated to lower surface of the membrane separating the upper from the lower chamber was counted after staining 0.5% crystal violet. Representative images (D) and quantification of results from 3 experiments, each performed in triplicate (E). Significant differences:*P<0.05; ** P<0.01; ***P<0.001 by two-way ANOVA for multiple comparisons with Tukey’s correction

FGF1 and FGF2, which activate FGFR2, are potent inducers of endothelial cell proliferation [48]. Consistently, FGF1 dose-dependently (0.3–9 ng/ml) promoted HUVEC proliferation in culture, whereas Ang2 (12.5–100 ng/ml) did not (Suppl. Figure 4). Additionally, Ang2 did not or minimally change FGF1-induced HUVEC proliferation (Fig. 6A and Suppl. Figure 4), despite Ang2 inhibiting Erk1/2 activation induced by FGF1 and FGF2 (Figs. 4 and 5). Inhibition of p-Erk 1/2 activity is often associated with reduced cell proliferation, but in endothelial cells inhibition of p-Erk activity has also been linked to reduced cell migration [49, 50].

We therefore tested the effects of Ang2 in a “wound-healing” assay in vitro, which relies on cell migration triggered by complex signals initiated by opening a space or “wound” in a cell monolayer [51]. In wounded monolayers of HUVEC, Ang2 (100 ng/ml) insignificantly affected spontaneous wound “healing” over 16 h, whereas FGF1 (10 ng/ml) time-dependently accelerated wound closure compared to the control (medium only). However, addition of Ang2 significantly reduced the wound-healing effect of FGF1 (Fig. 6B). Additionally, the Ang2 inhibitor, AMG386 (5 ng/ml) mitigated Ang2-induced inhibition of wound closure in the presence of FGF1, without affecting wound closure in medium only, with Ang2 or with FGF1 (Fig. 6C).

Using another test of cell motility, we examined HUVEC trans-well migration over 4 h incubation. The number of cells transmigrated in response to FGF1 (50 ng/ml) was increased compared to control. Ang2 (100 ng/ml) had no effect itself on cell transmigration, but significantly reduced FGF1-induced HUVEC transmigration (Fig. 6D, E). Thus, these experiments show that Ang2 serves as an inhibitor of endothelial cell movement induced by FGF1.

### Identification of Ang2 as an inhibitor of wound healing in the mouse

The “wound healing” experiments in vitro, showing that Ang2 is an inhibitor of endothelial cell migration and “wound” healing in response to FGF1, prompted examination of the effects of Ang2 in an experimental mouse model of dermal wound healing, in which the wounds are splinted to prevent rapid wound contraction [31]. This splinting renders the mouse model more dependent upon key steps in human wound repair, including hemostasis, inflammation, cell proliferation and remodeling that are critical to wound vascularization and epithelialization [3].

**Fig. 7:**
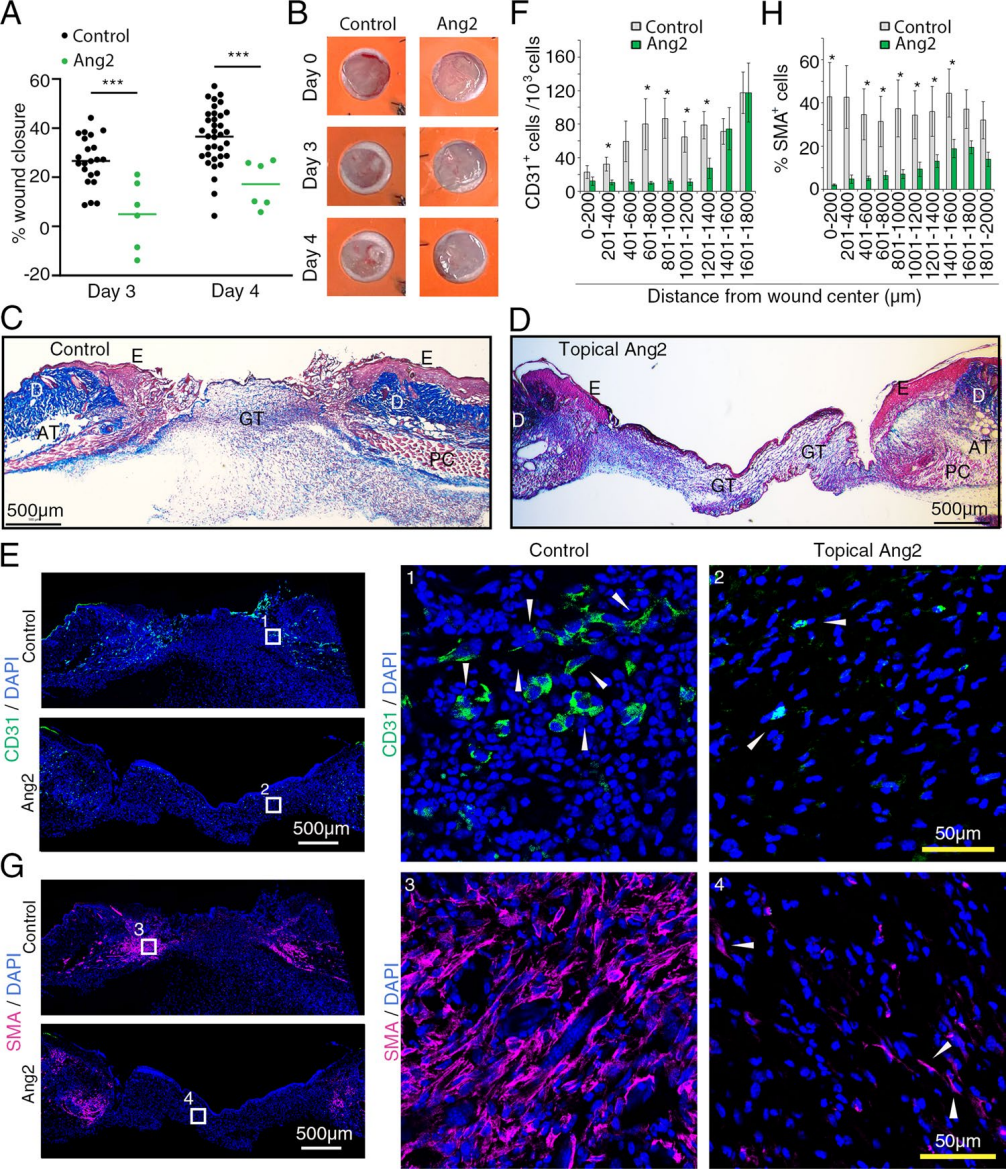
Topical Ang2 inhibits wound closure in mice. **A **Ang2 reduces wound closure on days 3 and 4 after wounding. Splinted wounds were treated topically with buffer only (PBS, 20 ml) or with Ang2 (1 mg/wound in 20 ml PBS) daily beginning on the day of wounding. Each dot represents individual wounds; control wounds, n=22 day 3 and n=35 day 4; treated wounds n=6 on day 3 and day 4. The results are presented as means from control and Ang2-treated wounds. ***P<0.001 by 2-way ANOVA for multiple comparisons with Sidak’s correction. **B **Representative images of splinted wounds treated topically with buffer (control) or Ang2 (1 mg/wound). The images reflect wound size change on days 3 and 4 from day 0 (day of wounding). **C, D **Histology of representative control (topical PBS only) and treated (topical Ang2) day 4 wounds stained with Masson’s trichrome. Epidermis (E, pink), dermis (D, dark blue), panniculus carnosus (PC, pink with typical morphology of the striated muscular layer), adipose tissue (AT, sparse cellularity area below the dermis) and GT (granulation tissue, blue). **E-H** Confocal immunofluorescence imaging (E and G) and quantification (F and H) of CD31^+^ cells or smooth muscle actin (SMA)^+^ cells in control (PBS only) and treated (topical Ang2) wounds. CD31^+^endothelial cells (E) and SMA^+^ cells without CD31^+^cell contact (G); DAPI staining detects cell nuclei. The rectangular areas (labeled 1-4) in the left panels are magnified on the right. Arrowheads point to CD31^+^ endothelial cells and SMA^+^ cells. Quantification of CD31^+^ cells (F) and SMA^+^ cells without CD31^+^cell contact (H) in control (n=4) and Ang2-treated (n=3) wounds in each 200 mm wound region from the wound center (0). The results reflect the means (± SEM); statistical significance by unpaired Student’s t-test. * P<0.05

First, we examined the effects of Ang2 on skin wound healing. Two wounds were generated on the back of individual mice; one wound was treated topically daily with Ang2, and the other received topical daily buffer only. Additional control wounds (buffer only) were generated in other mice (Suppl. Figure 5A). We found that topical Ang2 reduces wound healing on days 3 and 4 compared to controls (Fig. 7A, B, and Suppl. Figure 5B). Histology of representative wounds removed on day 4 (from day of wounding) and stained with Masson’s trichrome displayed typical features of cutaneous wounds at this time-point (Fig. 7C, D).

Immunofluorescence imaging showed the presence of CD31^+^ vascular structures and isolated endothelial cells in control and Ang2-treated wounds, particularly in areas away from the wound center (Fig. 7E, F). Quantitatively, the number of CD31^+^ cells was reduced in Ang2-treated wounds compared to control wounds and this difference was significant in areas at a distance ranging from 601 to 1400 μm from the wound center (Fig. 7F). Additionally, the Ang2-treated wounds contained fewer SMA^+^ cells (without CD31^+^ cell contact, broadly myofibroblasts and other mesenchymal cells) compared to control wounds, and this difference was significant in most areas of the wound, except in areas most distant (1601 to 2000 μm) from the wound center (Fig. 7G, H). However, the number of CD45^+^ inflammatory cells was similar in control and Ang2-treated wounds (Suppl. Figure 6 A, B). Thus, reduction of CD31^+^ endothelial cells/vascular structures and SMA^+^ mesenchymal-type cells characterizes delayed wounding induced by topical Ang2.

In additional experiments, we tested the effects of topical Ang2 in the presence of topical FGF2 (Trafermin). Topical Ang2 significantly inhibited wound healing on day 3 even in the presence of topical FGF2 (Suppl. Figure 7A, B). Of note, the current splinted wound healing assay did not reveal a wound healing activity of FGF2, despite previous studies showing that FGF2 (Trafermin) accelerates wound closure in other assay systems of acute and chronic skin wounds [54, 55]. Overall, these experiments show that topical Ang2 inhibits wound healing in the mouse.

Based on our results showing that Ang2 binds to and inhibits FGFR2 signaling and function, we hypothesized that endogenous Ang2, which is abundant in wounds [22, 23], functions as a natural inhibitor of wound repair by FGF/FGFR2. Although many growth factors, cytokines and chemokines and their receptors participate in regulating cutaneous wound healing [3, 22, 57, 58], FGF/FGFR signaling is required for the proper repair of cutaneous wounds [59]. Since AMG386 mitigates Ang2 inhibition of FGF1/FGFR-induced signaling in HUVEC and HEK293T cells in vitro (Figs. 4C and 5C), we now tested the effects of topical AMG386 in wound healing assays in vivo.

**Fig. 8:**
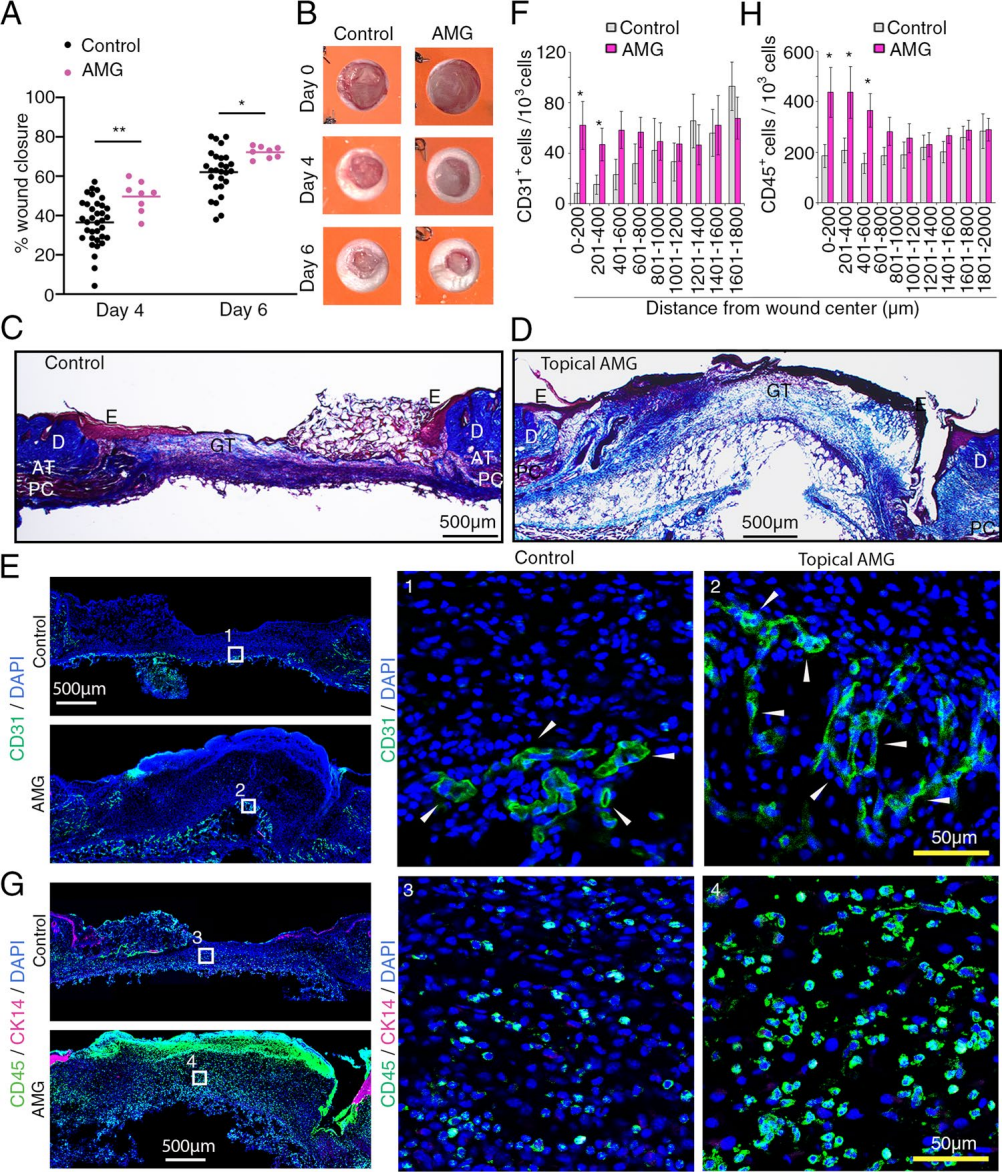
Topical AMG386 accelerates cutaneous wound healing. **A** Topical AMG386 (45 µg in 20 µl PBS /wound) applied to splinted cutaneous wounds immediately after wounding and then every other day beginning on day 2 significantly accelerates wound healing on day 4 and 6 compared to control (PBS, 20 µl). Each dot identifies individual wounds; control wounds: n=35 day 4 and n=27 day 6; treated wounds: n=8 on day 3 and day 6. The results are presented as means. * P<0.05, ** P<0.01; by 2-way ANOVA for multiple comparisons with Sidak’s correction. **B** Representative images of control wounds and wounds from mice treated with topical AMG386 photographed at the indicated time-points. **C, D** Histology of representative splinted wounds removed on day 4 from a control (C) (topical PBS only) and AMG386 treated (D) mouse; staining with Masson’s trichrome. **E-H** Confocal immunofluorescence imaging (E and G) and quantification (F and H) of CD31^+^ and CD45^+^ cells in control wounds and wounds treated with topical AMG386. The rectangular areas (labeled 1-4) in the left panels (E and G) are magnified on the right. Cytokeratin 14 (CK14) is visualized in magenta (G). Quantification of CD31^+^ (F) and CD45^+^ (H) cells in control (n=4) and AMG386-treated (n=4) wounds in each 200 µm wound region from the wound center (0). The results reflect the means (± SEM); statistical significance by unpaired Student’s t-test. * P<0.05

We applied AMG386 to the wound immediately after wounding and then every other day beginning on day 2. We found that AMG386 significantly accelerates wound healing on days 4 and 6 (Fig. 8A and B; Suppl. Figure 7C). In additional experiments we tested more directly the role of endogenous Ang2 in wound healing by using the Ang2-specific neutralizing antibody, REGN910/Nesvacumab [60], which does not bind to Ang1. We found that REGN910 significantly accelerated wound healing on day 4, and the magnitude of the effect was comparable to that of AMG386 (Suppl. Figure 7D). These results demonstrate that Ang2 functions as an endogenous inhibitor of cutaneous wound healing.

Representative histological features of cutaneous wounds removed on day 4 (from day of wounding) from control and topical AMG386-treated wounds are displayed in Fig. 8C and D. Immunofluorescence imaging (Fig. 8E-H) showed that wounds treated topically with AMG386 differ from control wounds in showing more numerous CD31^+^ endothelial cells (Fig. 8E, F) and CD45^+^ inflammatory cells (Fig. 8G, H) in the central regions of the wounds (CD31^+^ cells within the 0–400 μm, and CD45^+^ cells within the 0–600 μm areas from the wound center) (Fig. 8F, H). Imaging also showed a decrease of SMA^+^ cells in AMG386-treated wounds compared to control, particularly in wound regions located at 1001–1600 μm distance from wound center (Suppl. Figure 8 A, B). Thus, topical treatment with AMG386 promotes wound angiogenesis and inflammation.

We further examined vessel coverage with pericytes by quantifying the number of CD31^+^ endothelial cells with an adjacent (within 1 μm range) SMA^+^ cell (Suppl. Figure 8 C). This evaluation showed that Ang2-treated and AMG386-treated wounds display a similar pericyte vessel coverage (Suppl. Figure 8D, E). Finally, we looked for the presence of p-EGFR in the vasculature of cutaneous wounds removed on day 4, focusing in wound areas that most differentiate control wounds from wounds treated topically with Ang2 or AMG386 (Figs. 7F and 8F). We detected occasional CD31^+^ p-FGFR^+^ endothelial cells in control wounds, and virtually none in Ang-2 treated wounds (representative images, Suppl. Figure 9). In contrast, wounds treated with AMG386 contained more numerous p-FGFR^+^ cells, only some of which were CD31^+^ endothelial cells (Suppl. Figure 9). These results are consistent with a mechanistic role of FGFR modulation by Ang2 and AMG386 in cutaneous wound healing.

Overall, these results indicate that Ang2 is an inhibitor of wound healing, reducing angiogenesis and recruitment of myofibroblasts and other mesenchymal cells, and that blocking Ang2 by AMG386 promotes angiogenesis, recruitment of inflammatory cells and accelerates cutaneous wound healing.

## Discussion

In this study, we identified a previously unrecognized role of Ang2 in skin wound repair. Our results show that Ang2, an essential and context-dependent regulator of endothelial cells and vessels, operates as an endogenous inhibitor of angiogenesis during skin wound repair, and that Ang2 blockade with the peptibody AMG386 [42, 43, 61] promotes cutaneous wound healing. Biochemical experiments reveal that Ang2 is a previously unrecognized ligand of the tyrosine kinase receptor FGFR2 and an inhibitor of FGFR2 signaling induced by FGF in endothelial cells and other cells. These functions of Ang2 do not require the presence of the Ang2 receptor Tie2. In addition, functional studies in vitro show that Ang2 inhibits endothelial cell movement induced by FGF.

A wound healing function of Ang2 blockade has not, to our knowledge, been previously reported. However, it is known that Ang2 is induced after skin wounding, declines as wounds repair, is increased in diabetic wounds that often display impaired wound healing, and is elevated in the circulation of diabetic patients [23, 64, 65]. It is also known that the Ang2/Tie signaling pathway is critical to vascular development, regulation of angiogenesis and vascular remodeling, as well as regulation of vascular permeability, quiescence, and stability [16–18, 65, 66]. These distinct functions of Ang2 are highly context dependent and this contextuality has generally been attributed to Ang2 activation or inhibition of the Tie2 receptor [16, 19, 67, 68]. Mechanistically, Ang2/Tie2 actions are regulated by multiple factors, including the presence of Ang1 [16, 19, 67, 68]; the vascular endothelial protein tyrosine phosphatase (VE-PTP), which catalyzes the Tie2 dephosphorylation [69, 70]; the orphan receptor Tie1, which colocalizes with Tie2 at cell-to-cell contacts; and other incompletely defined variables [71]. Consistent with Ang1 modulating Ang2 in some settings, a form of recombinant Ang1, cartilage oligomeric matrix protein (COMP)-Ang1, was reported to promote wound healing through enhanced angiogenesis [72].

The current results provide a new insight into the contextual vascular effects of Ang2, distinct from the previous focus on Ang2/Tie2 interactions. The identification of Ang2 as an inhibitor of FGF1/FGFR2 signaling provides an opportunity for expanding current knowledge of the contextuality of Ang2 activities. FGF and FGFR2 play multiple roles in organ development, metabolism, tissue regeneration and inflammation [73]. FGF2 was the first endothelial cells growth factor identified [48], and subsequent studies have shown that FGF/FGFR2 signaling induces angiogenesis by promoting endothelial cell proliferation, migration, and differentiation [74]. In the context of tissue repair, FGF2 deficiency [55] or endothelial deficiency of FGFR1 and FGFR2 [59] significantly impair neovascularization and cutaneous wound repair. Thus, despite redundancy of angiogenic factors, FGF2 and FGFR1/2 play an essential role in promoting angiogenesis during skin wound healing. Topical FGF2 has been approved in Japan for treatment of wounds since 2001.

Ang2 is expressed in endothelial cells and in fibroblasts/myofibroblasts in human surgical wounds [22, 23]. Our current results indicate that Ang2 is an endogenous inhibitor of FGF/FGFR-induced angiogenesis and skin wound repair, and that blocking this inhibitory function of Ang2 with AMG386 or the Ang2-specific neutralizing antibody REGN910/Nesvacumab and potentially other Ang2 inhibitors promotes wound angiogenesis and repair. It remains unclear, however, if Ang2 inhibition of FGFR2 signaling is the mechanism by which Ang2 functions as an endogenous inhibitor of wound healing. To address this limitation, a mouse model of conditional FGFR2 knockout or other tools to block FGFR2 signaling, such as small molecules and specific antibodies, will be required. The experimental data in vitro presented here would predict that blockade of Ang2 would be ineffective at improving wound healing when the target FGFR2 is functionally impaired. AMG386 has been extensively tested in human cancer clinical trials and has been found to be well tolerated [42, 61] and REGN910/Nesvacumab has undergone initial testing in patients [60]. The current results support the testing of AMG386 as a treatment for skin wound healing. Despite advances, there is much need to identify new targeted therapeutics to promote wound repair [7], particularly in the elderly and diabetic populations.

In summary, our findings demonstrate that Ang2 is an endogenous inhibitor of skin wound healing and that Ang2 blockade accelerates wound healing. Thus, intervention by Ang2 blockade may provide a new evidence-based therapeutic option for promoting wound repair.

## Electronic supplementary material

Below is the link to the electronic supplementary material.

Supplementary Material 1

## Data Availability

No datasets were generated or analysed during the current study.
