# Supplementary material for: Angiopoietin-2 binds to FGFR2, inhibits FGF-FGFR2 signaling, and delays cutaneous wound healing by inhibiting wound angiogenesis
Source: Angiogenesis. 2025 Aug 5;28(4):43. doi: 10.1007/s10456-025-09988-2 (PMC12325526; doi:10.1007/s10456-025-09988-2)
Supplement: Supplementary file 1 — Supplementary Material 1 [file 10456_2025_9988_MOESM1_ESM.pdf]

© Springer Nature

Insert Element
Section Style
Insert Citation
Edit
AQ List
ToC
Comments

[illegible]

Aff1 Laboratory of Cellular Oncology, Center for Cancer Research, National Cancer Institute, National Institutes of Health, Bethesda, MD, 20892, USA

Aff2 Laboratory of Cell Biology, Center for Cancer Research, National Cancer Institute, National Institutes of Health, Bethesda, MD, 20892, USA

Aff3 Experimental Retrovirology Section, HIV & AIDS Malignancy Branch, Center for Cancer Research, National Cancer Institute, National Institutes of Health, Bethesda, MD, 20892, USA

Aff4 Biophysics Resource, Center for Structural Biology, National Cancer Institute, Frederick, MD, 21702, USA

Abstract

Wound healing is an essential repair process, and impaired wound healing is a common and sometimes debilitating medical problem. Despite advances in wound healing approaches, optimal management strategies are lacking, partly due to an incomplete understanding of the complex pathophysiology of this process. Here we show that Ang2, a previously known ligand for the Tie2 receptor, also binds to fibroblast growth factor receptor 2 (FGFR2) independently of Tie2 and attenuates FGF/FGFR2 signaling in endothelial cells. Functionally, Ang2 inhibits endothelial cell migration induced by FGF. In mouse, topical Ang2 delays the healing of skin wounds, associated with reduced wound angiogenesis and recruitment of mesenchymal-type cells. Additionally, topical AMG386, a blocker of Ang1/Ang2 binding to Tie2 and systemic REGN910, a blocker of Ang2 binding to Tie2, accelerate wound repair, associated with increased wound angiogenesis and recruitment of inflammatory cells. These results identify the tyrosine kinase FGFR2 as a previously unrecognized Ang2 receptor, explaining some of the context-dependent functions of Ang2 in endothelial cells. Since Ang2 is

DOI: 10.1007/s10456-025-09988-2

Cookie Settings Privacy Policy

undergoing marked changes in gene expression, signaling and phenotype leading to cell migration, proliferation, and differentiation. If successful,

© Springer Nature

Insert Element

Section Style

Insert Citation

IA

DOI: 10.1007/s10456-025-09988-2

Cookie Settings Privacy Policy
